# Meniscal extrusion increases with age: Decade‐specific magnetic resonance imaging reference values

**DOI:** 10.1002/jeo2.70839

**Published:** 2026-07-13

**Authors:** Riccardo Compagnoni, Rossella Ravaglia, Matteo Parmigiani, Alessio Maione, Filippo Pierfrancesco Calanna, Paolo Ferrua, Pietro Simone Randelli

**Affiliations:** ^1^ Department of Biomedical, Surgical and Dental Sciences Università degli Studi di Milano Milan Italy; ^2^ 2 ° Clinica Ortopedica, ASST Centro Specialistico Ortopedico Traumatologico Gaetano Pini‐CTO Milan Italy; ^3^ University of Milan, School of Specialization in Orthopedics and Traumatology Milan Italy; ^4^ 1° Clinica Ortopedica, ASST Centro Specialistico Ortopedico Traumatologico Gaetano Pini‐CTO Milan Italy; ^5^ Week Surgery, ASST Centro Specialistico Ortopedico Traumatologico Gaetano Pini‐CTO Milan Italy; ^6^ Department of Biomedical Sciences for Health Laboratory of Applied Biomechanics Università degli Studi di Milano Milan Italy

**Keywords:** aging, knee MRI, meniscal extrusion, meniscal extrusion index, normative values

## Abstract

**Purpose:**

To establish decade‐specific magnetic resonance imaging (MRI)‐based reference values for the medial meniscal extrusion index (MEI) in adults without significant structural knee pathology and to assess associations with age and sex. The hypothesis was that decade‐specific MEI values would increase with age, without meaningful sex‐related differences, while remaining low in knees without significant structural abnormalities on MRI.

**Methods:**

In June 2025, knee MRI examinations from a single tertiary orthopaedic centre were retrospectively screened until 500 eligible knees were identified (100 per decade, ages 20–69; balanced by sex). Inclusion criteria were age 20–69 years, 1.5‐T MRI, and no definite/moderate osteoarthritis on MRI. Exclusion criteria included root, radial or complex meniscal tears; significant focal chondral lesions; ligament injury; discoid meniscus; prior knee surgery; inflammatory disease; evident anatomical abnormalities; and marked bone marrow edema. MEI was measured on coronal images as the ratio of extruded medial meniscus to total medial meniscal width. Linear regression tested the association between age and MEI, including an age × sex interaction term. Reference values were reported per decade as mean, standard deviation (SD) and mean + 1 SD and + 2 SD. Interobserver reliability was assessed using the intraclass correlation coefficient (ICC).

**Results:**

MEI was positively associated with age (*β* = 0.0019 per year, *R* ^2^ = 0.072, *p* < 0.001). No significant age × sex interaction was detected. Mean MEI increased from 0.041 in the 20–29 decade to 0.122 in the 60–69 decade. The mean + 1 SD threshold approached a 20% MEI cut‐off in the 50–59 decade and exceeded it in the 60–69 decade. Interobserver reliability was excellent (ICC ≈ 0.82).

**Conclusions:**

MEI increased progressively with age in knees without significant structural pathology on MRI, with no meaningful sex‐related differences. Decade‐specific MEI reference intervals may support age‐adjusted interpretation of isolated medial meniscal extrusion, particularly in older adults, while reinforcing its role as an adjunctive imaging finding.

**Level of Evidence:**

Level III, retrospective cohort study.

## Graphical abstract

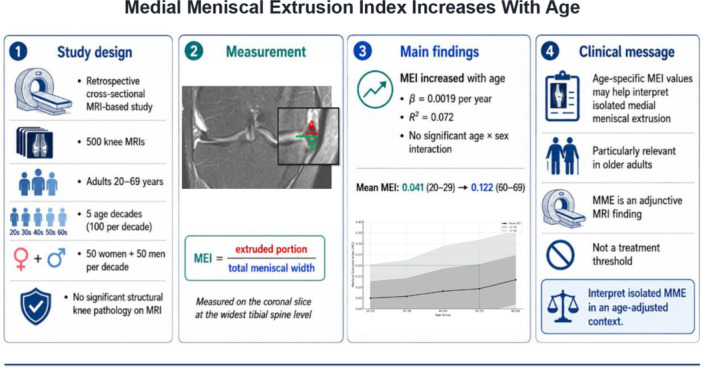

**Abbreviations**
BMI: body mass index
CI: confidence interval
ICC: intraclass correlation coefficient
IRCCS: Istituto di Ricovero e Cura a Carattere Scientifico
LCL: lateral collateral ligament
MCL: medial collateral ligament
ME: meniscal extrusion
MEI: meniscal extrusion index
MOAKS: MRI osteoarthritis knee score
MRI: magnetic resonance imaging
PD: proton density
SD: standard deviation
STIR: short tau inversion recovery

## INTRODUCTION

Medial meniscal extrusion (MME), defined as the radial displacement of the medial meniscal tissue beyond the tibial plateau, is a recognised indicator of meniscal dysfunction and increased tibiofemoral contact pressures [7, 8]. It reflects a loss of the meniscus's ability to dissipate hoop stresses, potentially accelerating degenerative changes in the knee [11, 12, 16].

Several classification systems have been proposed to define and quantify meniscal extrusion, based on absolute measurements [7], relative indices [5] or qualitative assessments [17]. However, no universal consensus exists on which criteria should be applied, and methodological differences make it difficult to compare results across studies [9].

Recent studies in healthy volunteers using magnetic resonance imaging (MRI) and ultrasound have shown a significant increase in medial meniscus extrusion with advancing age and higher body mass index (BMI) (*p* < 0.001) [1, 10, 18]. Negishi et al. reported that MME was present in nearly 98% of asymptomatic elderly subjects, highlighting its potential as a paraphysiological finding [15].

However, no large‐scale study has yet provided decade‐specific MEI reference values stratified by age and sex in adults without significant structural knee pathology on MRI. This lack of reference data limits the ability to contextualise the magnitude of isolated MME, particularly in older adults, where mild extrusion may be observed even in the absence of relevant meniscal, chondral or ligamentous abnormalities.

Therefore, the aim of this study was to establish decade‐specific meniscal extrusion index (MEI) reference values in adults aged 20–69 years without significant structural knee pathology on MRI, and to assess potential associations with age and sex.

The hypothesis was that decade‐specific MEI values would show a progressive age‐related increase, without meaningful sex‐related differences, while remaining low in knees without significant structural abnormalities on MRI.

## MATERIALS AND METHODS

This retrospective cross‐sectional MRI‐based study was performed at a large orthopaedic university hospital and approved by the local ethical committee (authorisation number: Fondazione IRCCS Ca’ Granda Ospedale Maggiore Policlinico–Milano Area 2, Lombardia, Milan, n°394_2019bis, Milan, 08.05.2019).

All knee MRI scans performed at the study hospital were reviewed retrospectively, starting from June 2025 and proceeding backwards in time until a total of 500 eligible examinations were identified. To better characterise age‐related changes in MME across the adult lifespan, subjects were stratified into age decades. The evaluations were conducted using imaging data acquired with the same 1.5 Tesla MRI scanner (MAGNETOM Espree, Siemens Healthineers, Erlangen, Germany).

Proton density (PD), short tau inversion recovery (STIR) and T2 fat‐saturated sequences were analysed on the coronal plane.

Inclusion criteria:

- Age between 20 and 69 years
- MRI performed on the same 1.5 T scanner
- No definite/moderate osteoarthritis on MRI, defined using the MRI osteoarthritis knee score (MOAKS) as: [13]

− cartilage loss involving <10% of any tibiofemoral subregion and no full‐thickness cartilage loss;
− none or small osteophytes (MOAKS ≤ 1) in all marginal locations;
− none or small bone‐marrow lesions involving <33% of any subregion (MOAKS ≤ 1).

Exclusion Criteria:

- Medial meniscal tears expected to impair hoop function, including root, radial, complex, flap tears, displaced tears and meniscal maceration. Isolated horizontal tears without radial/root components, displaced fragments or meniscal maceration were not considered exclusion criteria.
- Significant focal chondral lesions, defined as cartilage loss involving ≥10% of any tibiofemoral subregion or any full‐thickness cartilage loss.
- Central or peripheral ligament injuries, including anterior cruciate ligament, posterior cruciate ligament, medial collateral ligament and lateral collateral ligament injuries.
- Discoid meniscus
- Rheumatic or inflammatory joint diseases or synovitis
- History of previous knee surgery
- Evident anatomical abnormalities on MRI, including marked posttraumatic deformity, congenital deformity, severe dysplasia or gross structural deformity visible on standard knee MRI.

**Figure 1:**
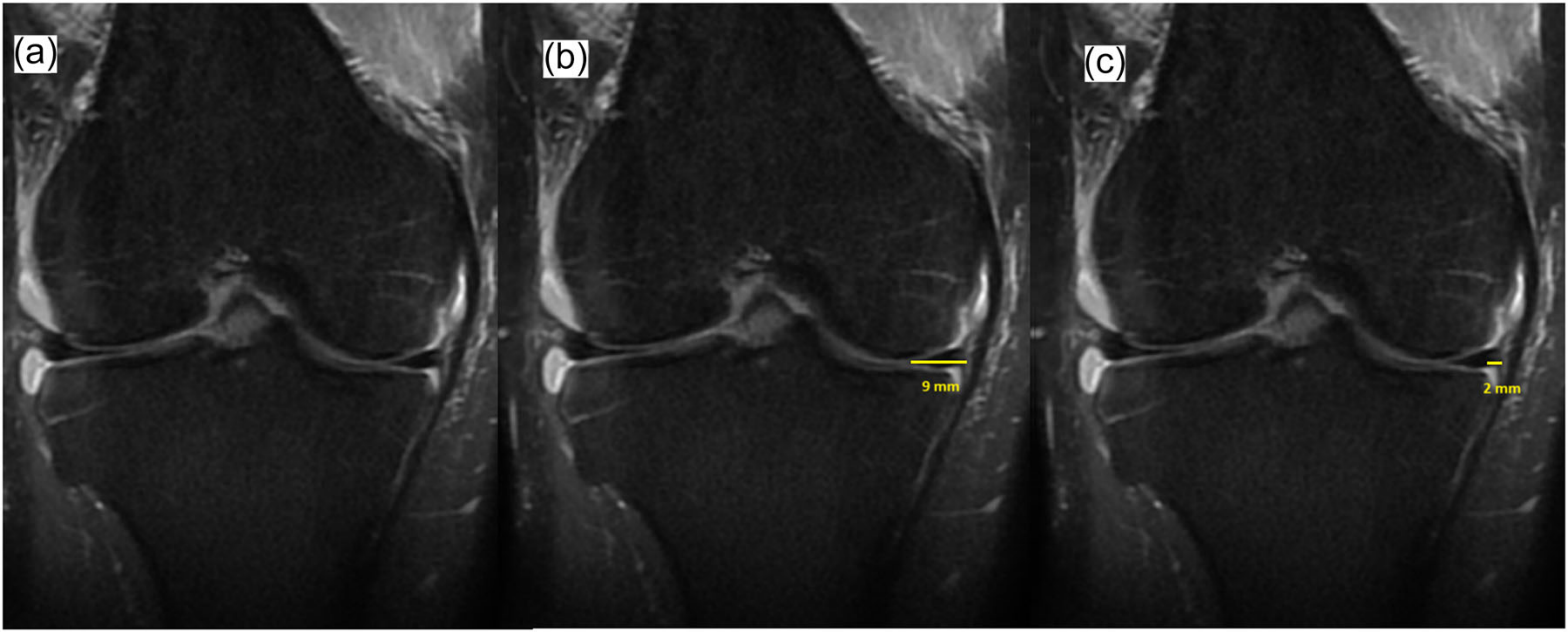
(a) Coronal MRI with a medial meniscal extrusion; (b) medial meniscal width; (c) overhanging portion of the meniscus. MRI, magnetic resonance imaging.

The measurement slice was selected as the coronal plane showing the widest point of the tibial spines, as described by Costa et al. [7]. This slice was selected because the tibial spines provide a reproducible anatomical landmark for standardised coronal‐plane assessment, reducing operator‐dependent selection of the slice with maximal apparent extrusion. On this slice, absolute MME was measured as the distance between the medial edge of the tibial plateau and the outer margin of the medial meniscus. Total medial meniscal width was measured on the same line as the sum of the extruded portion and the nonextruded intraarticular portion of the meniscus, as shown in Figure 1 [11].

Absolute MME and total medial meniscal width were recorded in millimetres.

The MEI was calculated by determining the ratio between the extruded portion of the meniscus and the total width of the meniscus [5].

In the present study, MEI was selected as the primary measure because it expresses extrusion as a proportion of total meniscal width, thereby accounting for interindividual differences in meniscal size. Absolute MME values in millimetres were also reported to improve clinical interpretability and allow comparison with previous studies based on absolute displacement thresholds.

MRI examinations were screened in reverse chronological order and were not randomised before measurement. Raters were not formally blinded to age or sex.

The program used for the measurement was Xero Viewer (Agfa Healthcare).

Interobserver reliability for MEI measurements was assessed between two independent raters using a two‐way random‐effects intraclass correlation coefficient [ICC(2,1)], absolute agreement definition. The standard error of measurement (SEM) for MEI was calculated as SD × √(1 − ICC).

### Power analysis

A priori sample size calculation was conducted to determine the minimum number of subjects required to detect a statistically significant relationship between age and the MEI, using a linear regression model. Assuming a significance level (*α*) of 0.05, a statistical power (1–β) of 80% and an a priori conservative small effect size (*f* ^2^ = 0.035), the required sample size was calculated to be approximately 288 subjects.

To ensure sufficient power for secondary subgroup analyses and account for potential exclusions, the total sample size was set at 500 participants, distributed evenly across five age decades (20–29, 30–39, 40–49, 50–59, and 60–69 years), with equal representation of males and females in each group. With this sample size, the power to detect the predefined effect size exceeds 99%.

### Statistical approach

Descriptive statistics will be reported as means and standard deviations (SDs) for continuous variables, and as counts and percentages for categorical variables. The normality of data distributions will be evaluated using the Shapiro–Wilk test and visual inspection of histograms and Q–Q plots.

The association between age and MEI will be assessed using simple linear regression, with MEI as the dependent variable and age as the independent variable. The *β* coefficient will quantify the annual change in MEI. Statistical significance will be considered at *p* < 0.05. Model performance will be evaluated using the coefficient of determination (*R* ^2^).

To explore potential differences in MEI progression between sexes, an interaction term (age × sex) was included in the linear regression model. Sex was coded as a binary variable (0 = male, 1 = female), and interaction significance was assessed to determine whether stratified analysis was warranted.

To provide normative data for clinical reference, the study population was stratified by age decades (20–29, 30–39, 40–49, 50–59, 60–69 years). For each group, the mean MEI and SD were calculated. Reference thresholds were defined as the group mean plus one and two SDs (mean +1 SD and mean + 2 SD).

## RESULTS

A total of 1357 knee MRI scans were reviewed for eligibility. After the application of predefined inclusion and exclusion criteria, 500 MRI scans were included in the final analysis.

Interobserver reliability for MEI measurement was excellent, with an ICC(2,1) of 0.819 (95% CI: 0.787–0.846). The SEM for MEI was 0.044.

The demographic characteristics of the included sample are summarised in Table 1. The population consisted of male and female patients between 20 and 69 years of age. The distribution across age groups was balanced, allowing for reliable analysis of the age‐related progression of meniscal extrusion.

**Table 1:** Demographic data of the included population.

Decade	Sex	*n*°	Mean age	SD age
20–29	F	50	24.7	2.4
20–29	M	50	24.3	3.1
30–39	F	50	34.2	2.7
30–39	M	50	34.2	2.7
40–49	F	50	44.9	3.0
40–49	M	50	44.2	3.0
50–59	F	50	54.5	3.1
50–59	M	50	54.6	2.7
60–69	F	50	63.8	2.4
60–69	M	50	64.4	2.7
Total	—	500	—	—

Abbreviations: F, female; M, male; SD, standard deviation.

Across the entire cohort, absolute MME values ranged from 0 to 4 mm, while MEI values ranged from 0 to 0.444.

**Figure 2:**
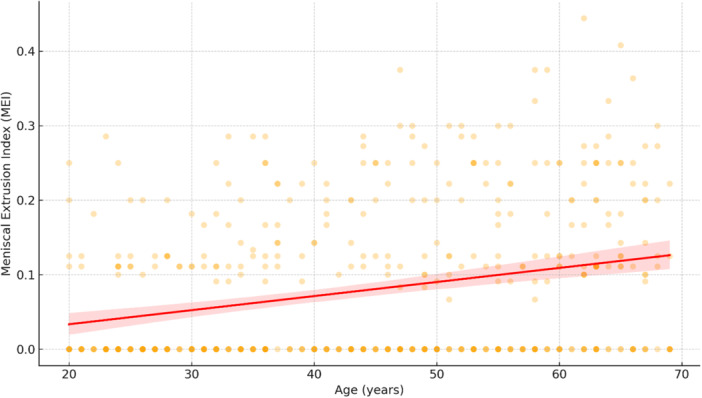
Scatterplot and linear regression model showing the relationship between age and meniscal extrusion index (MEI). Each dot represents an individual subject.

The regression model revealed a significant positive association between age and MEI (*β* = 0.0019, *p* < 0.001), indicating that the MEI increased by approximately 0.19% per year of age. The model was statistically significant (*F*(1, 498) = 38.5, *p* < 0.001), with an *R* ^2^ of 0.072, meaning that age explained 7.2% of the variability in MEI values. The regression line and the distribution of MEI values across the age range are illustrated in Figure 2.

The regression model including the age × sex interaction did not show a statistically significant interaction effect (*p* = 0.18), indicating that the rate of increase in MEI with age was comparable between males and females.

To provide a reference framework for the age‐related distribution of the MEI, values were analysed according to age decades. The results showed a progressive increase in the mean MEI with advancing age, ranging from 0.04 in the 20–29 decade to 0.12 in the 60–69 group.

Table 2 reports the mean MEI values, SDs and calculated upper limits corresponding to one and two SDs above the mean.

**Table 2:** Decade‐specific MEI and absolute MME values, with MEI reference limits defined by +1 and +2 SDs.

Age decade	*n*	Mean MEI	SD MEI	Mean MME, mm	SD MME, mm	MEI mean + 1 SD	MEI mean + 2 SD
20–29	100	0.041	0.071	0.36	0.63	0.112	0.183
30–39	100	0.065	0.088	0.57	0.76	0.153	0.241
40–49	100	0.082	0.106	0.70	0.95	0.188	0.284
50–59	100	0.092	0.112	0.83	1.01	0.204	0.317
60–69	100	0.122	0.111	1.12	1.06	0.233	0.344

Abbreviations: MEI, meniscal extrusion increases; MME, medial meniscal extrusion; SD, standard deviation.

**Figure 3:**
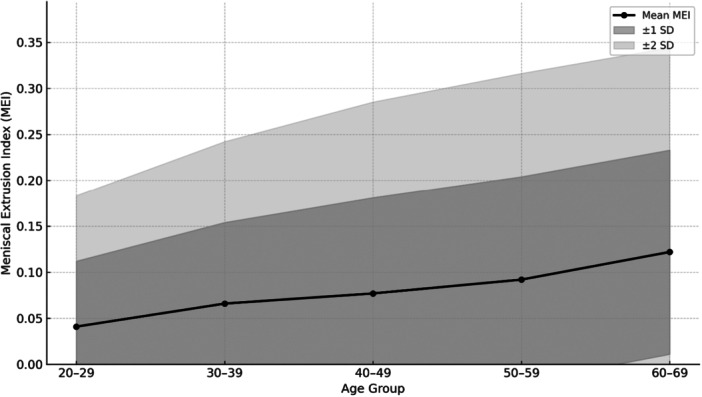
Mean meniscal extrusion index (MEI) by age decade with bands indicating ±1 standard deviation (light grey) and ±2 standard deviations (lightest grey). The MEI shows a progressive increase with age, with greater variability in older age groups.

A graphical representation of the mean MEI values with ±1 and ±2 SD bands across age decades is shown in Figure 3. The plot highlights a gradual upward trend in both the mean and variability of MEI with increasing age, with wider dispersion observed in older groups.

**Figure 4:**
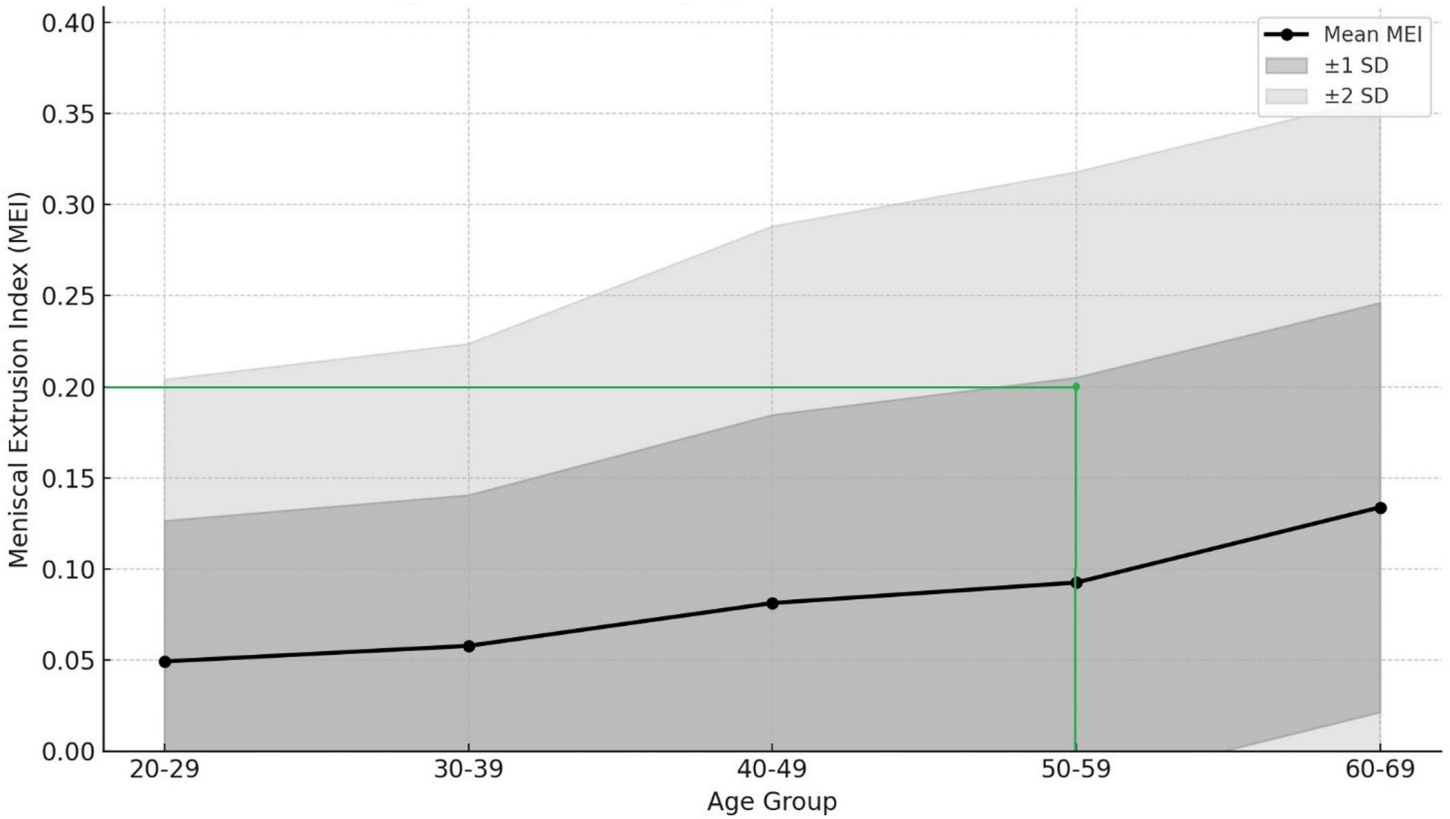
Mean meniscal extrusion index (MEI) by age decade with ±1 and ±2 standard deviation (SD) bands. The horizontal green line represents the 20% MEI threshold proposed by Compagnoni et al. [5]. The vertical green line marks the decade in which the mean +1 SD first intersects this threshold.

According to the classification proposed by Compagnoni et al. [6], MEI values <20% are considered para‐physiological. In the present cohort, the mean + 1 SD threshold for the 50–59 age group approaches this 20% cut‐off, while in the 60–69 decade, the mean + 1 SD already exceeds it (Figure 4).

## DISCUSSION

This study provides, for the first time, normative reference values for meniscal extrusion in adult knees aged 20–69 years, stratified by decade. The results confirm a significant, progressive increase in MEI with advancing age, even in the absence of relevant knee pathology. The positive slope of the regression model (*β* = 0.0020) indicates an average increase of approximately 0.20% per year of age. The reference thresholds defined as mean + 1 SD and mean + 2 SD for each decade may assist clinicians supporting age‐adjusted interpretation of isolated MME. In addition, interobserver reliability for MEI measurements was excellent [ICC(2,1) = 0.819], supporting the reproducibility of this method in routine MRI analysis.

Previous studies have primarily investigated meniscal extrusion in populations with meniscal or osteoarticular pathology [2, 5, 10, 12, 14]. For example, Costa et al. [7] and Barreira et al. [2]. proposed absolute displacement cut‐offs (≥ 3 mm) measured on MRI, but these thresholds were derived from pathological cohorts and do not account for age‐related variation. More recent work by Compagnoni et al. [5]. introduced the MEI as a percentage‐based measurement, which offers the advantage of accounting for individual anatomical variation. Perelli et al. [17]. proposed a qualitative classification that also acknowledges the possibility of low‐grade meniscal extrusion in knees without major structural abnormalities.

In the present study, MEI was selected as the primary outcome because it expresses extrusion relative to total meniscal width, thereby reducing the influence of interindividual differences in meniscal size. This may be particularly relevant when comparing subjects across different ages and sexes.

Studies in asymptomatic individuals confirm that MME increases with age. Jungmann et al. [9]. found a significant correlation between age, BMI and medial meniscus extrusion in healthy volunteers, while Shimozaki et al. [18]. demonstrated increased extrusion under loading conditions in nonpathological knees. Negishi et al. [15]. reported that MME was present in nearly all elderly asymptomatic subjects, suggesting that some extrusion may represent an age‐related finding. The results expand upon these findings by providing decade‐specific MEI values and quantifying the rate of increase across the adult lifespan.

No significant age × sex interaction was detected, indicating that the rate of MEI increase with age is comparable in men and women. This finding is consistent with previous literature, where sex‐related differences were not evident when values were expressed as a percentage of meniscal width, as reported by Bloecker et al. [3].

From a clinical perspective, the present findings suggest that isolated MME should be interpreted in an age‐adjusted context, particularly in older adults and when no relevant meniscal, chondral or ligamentous abnormalities are present. In this cohort, mean MEI and absolute MME values increased with age but remained low across all decades.

Applying absolute displacement cut‐offs without accounting for age‐related variation may lead to overinterpretation of isolated MME.

The decade‐specific reference intervals reported here may help contextualise MEI measurements within the expected age‐related spectrum. This is particularly relevant in the assessment of incidental meniscal findings in middle‐aged or elderly patients, as highlighted by the MenIN consensus [9], which underlined the current heterogeneity in classification and interpretation.

In the classification proposed by Compagnoni et al. [5], a MEI < 20% is considered likely para‐physiological, 20%–40% represents a ‘grey zone’, and > 40% is typically associated with lesions impairing meniscal function.

In the present cohort, the mean +1 SD threshold approached the 20% cut‐off in the 50–59 decade and exceeded it in the 60–69 decade. This finding suggests that caution is warranted when applying a single 20% threshold to older adults, as isolated MEI values slightly above this threshold may occur even in knees without significant structural pathology on MRI.

The strengths of this study include its large sample size (*n* = 500), strict inclusion/exclusion criteria ensuring the absence of significant knee pathology, and balanced distribution across age decades and sex. The use of a percentage‐based index (MEI) minimises the effect of individual anatomical variation compared to absolute displacement measures. In order to characterise age‐related MME, only knees without relevant meniscal, chondral or ligamentous injury, previous surgery, joint space narrowing or marked bone marrow edema were selected, as the latter is typically associated with intra‐articular pathology rather than physiological changes [6]. Furthermore, the excellent interobserver reliability observed here supports the reproducibility of MEI assessment in clinical and research settings.

Several limitations should be acknowledged. This was a retrospective, single‐centre study, which may limit generalisability.

Another limitation is that MRI scans were obtained in the non‐weight‐bearing neutral position. Weight‐bearing imaging, including dynamic ultrasound, was not available and could complement static MRI assessment in future studies [4, 19].

In addition, full‐leg standing radiographs were not available; therefore, lower‐limb alignment could not be assessed. BMI, physical activity level and other lifestyle factors were not consistently available and were therefore not included in the regression model. Their absence may partly explain the low *R* ^2^ observed in the age‐based model, indicating that age accounts for only a limited proportion of MEI variability. Future studies should include these variables to better define their independent contribution to MME.

Future research should aim to validate these decade‐specific MEI reference values in multicenter, prospective cohorts.

## CONCLUSION

This study provides decade‐specific MRI‐based reference values for MEI in adults aged 20–69 years without significant structural knee pathology on MRI. The present findings confirm the hypothesis of a progressive age‐related increase in MEI, with no meaningful sex‐related differences observed. By offering reference intervals based on SDs for each decade, this work may support a more age‐adjusted interpretation of MME, particularly in older adults, while reinforcing its role as an adjunctive imaging finding within the overall clinical and MRI assessment.

## AUTHOR CONTRIBUTIONS

Riccardo Compagnoni conceived the study. Rossella Ravaglia performed the statistical analysis, collected the data and contributed to drafting the manuscript. Matteo Parmigiani collected the data. Filippo Pierfrancesco Calanna and Alessio Maione contributed to the study concept and to drafting the manuscript. Paolo Ferrua critically revised the manuscript. Pietro Simone Randelli supervised and directed the study. All authors read and approved the final manuscript.

## FUNDING INFORMATION

The authors have no funding to report.

## CONFLICT OF INTEREST STATEMENT

Pietro Randelli serves in a consulting/advisory role for MicroPort Orthopedics Inc. and Medacta International SA. No payments were received in relation to the submitted work. The remaining authors declare that they have no conflicts of interest related to this manuscript.

## ETHICS STATEMENT

The study was approved by the local ethics committee (Fondazione IRCCS Ca' Granda Ospedale Maggiore Policlinico–Milano Area 2, Lombardia, Milan; authorisation number 394_2019bis, 08.05.2019).

## Data Availability

The data that support the findings of this study are available from the corresponding author upon reasonable request. Data are not publicly available due to privacy and ethical restrictions.
